# P-1194. Comparison of COVID-19 inpatient burden in hospitalized children age < 5 years, by SARS-CoV-2 variant

**DOI:** 10.1093/ofid/ofae631.1378

**Published:** 2025-01-29

**Authors:** Kathleen M Andersen, Thomas M Porter, Maya Reimbaeva, Maria McColgan, Santiago M C Lopez

**Affiliations:** Pfizer Inc., New York, New York; Pfizer Inc., New York, New York; Pfizer, Inc., Groton, Connecticut; Pfizer Inc., New York, New York; Pfizer Inc, Collegeville, Pennsylvania

## Abstract

**Background:**

COVID-19 disease severity has fluctuated with emergence of variants. To investigate disease severity in children, we analyzed clinical resource utilization among children hospitalized for COVID-19 during different variant periods in the United States.

**Figure:**
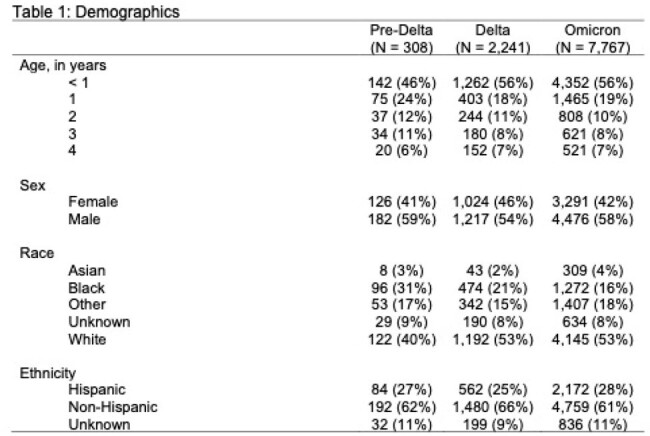

**Methods:**

We constructed a cohort of children age < 5 years hospitalized for COVID-19 (ICD-10-CM code U07.1 “COVID-19”) using PINC AI Healthcare Data, a hospital-based real world data source from the United States. Hospitalizations in pre-Delta (April - June 2021), Delta (July - December 2021) or Omicron (January 2022 – July 2023) variant periods were included. We examined pre-existing conditions, hospitalization length of stay (LOS), supplemental oxygen (O2), admission to and number of days in the intensive care unit (ICU) and receipt of invasive mechanical ventilation (IMV). P-values for 3-way comparisons were calculated using Chi-square tests for proportions and Kruskal-Wallis for medians.

**Figure:**
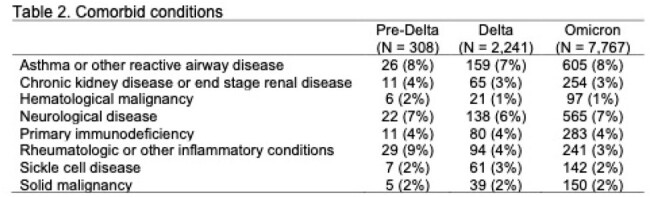

**Results:**

Of 10,316 children hospitalized for COVID-19, the majority occurred in the Omicron period (n=7,767, 75%) followed by Delta (2,241; 22%) and pre-Delta (308; 3%) periods (Table 1). The age, sex, race and ethnicity distribution of cases was similar between Delta and Omicron eras. Rheumatologic and other inflammatory conditions were more prevalent during pre-Delta; otherwise, the prevalence of pre-existing health conditions was generally similar across each variant wave (Table 2). Among the following outcomes, there were no significant differences across the variants: supplemental O2 (13% pre-Delta, 16% Delta, 18% Omicron, p = 0.06), LOS (median 2 days), proportion requiring ICU care (20-21%, p = 0.77), days in ICU care (median 1 pre-Delta, 2 Delta and Omicron) and proportion requiring IMV (6-8%, p = 0.35) (Table 3). Deaths were more common in the Delta (0.9%) than pre-Delta (0.7%) or Omicron (0.4%) eras.

**Figure:**
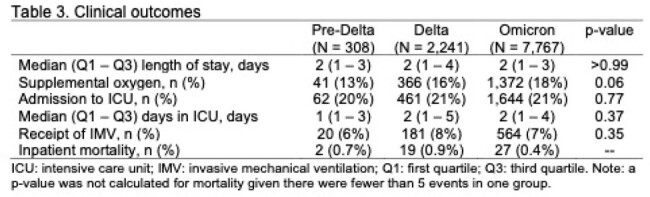

**Conclusion:**

Similar disease severity was observed among children age < 5 years hospitalized for COVID-19 during pre-Delta, Delta and Omicron periods. Although in-hospital death was more common with Delta, Omicron led to increased hospitalizations, highlighting the importance of continued preventative measures to prevent SARS-CoV-2 infection in children, including routine immunization.

**Disclosures:**

**Kathleen M. Andersen, PhD MSc**, Pfizer Inc.: Employee|Pfizer Inc.: Stocks/Bonds (Public Company) **Thomas M. Porter, MPH**, Pfizer Inc.: Employee|Pfizer Inc.: Stocks/Bonds (Public Company) **Maya Reimbaeva, MS**, Pfizer Inc.: Employee|Pfizer Inc.: Stocks/Bonds (Public Company) **Maria McColgan, MD**, Pfizer Inc.: Employee|Pfizer Inc.: Stocks/Bonds (Public Company) **Santiago M.C. Lopez, MD**, Pfizer Inc.: Employee|Pfizer Inc.: Stocks/Bonds (Public Company)

